# A systematic review on the utility of non-invasive electrophysiological assessment in evaluating for intra uterine growth restriction

**DOI:** 10.1186/s12884-019-2357-9

**Published:** 2019-07-05

**Authors:** Vinayak Smith, Amrish Nair, Ritesh Warty, Joel Arun Sursas, Fabricio da Silva Costa, Euan Morrison Wallace

**Affiliations:** 1 Department of Obstetrics and Gynaecology, Monash University, 252 Clayton Road, Clayton, Victoria 3168 Australia; 2 Biorithm Pte Ltd, 81 Ayer Rajah Crescent 03-53, Singapore, 139967 Singapore; 3 Department of Gynecology and Obstetrics, Ribeirão Preto Medical School, Ribeirão Preto, São Paulo Brazil

**Keywords:** Fetal electrocardiogram, Fetal magnetocardiogram, Cardiac time intervals, Short term variability, Long term variability, Intra uterine growth restriction, PRSA

## Abstract

**Background:**

Non-invasive electrophysiological assessment (NIEA) is an evolving area in fetal surveillance and is attracting increasing research interest. There is however, limited data outlining its utility in evaluating intra uterine growth restriction (IUGR). The objective of this study was to carry out a systematic review to outline the utility of NIEA parameters in evaluating IUGR.

**Methods:**

A systematic review of peer reviewed literature was performed, searching PUBMED, Ovid MEDLINE and EMBASE. The outcomes of interest included NIEA parameters [P wave duration, PR interval, QRS duration, QT interval, T/QRS ratio, short term variability (STV) and long term variability (LTV)] and a descriptive summary of relevant studies as well.

**Results:**

Sixteen studies were identified as suitable for inclusion. The utility of NIEA parameters were investigated in tabular form. In particular, QRS and QT duration, T/QRS ratio, STV and PRSA analysis displayed utility and merit further consideration in evaluating for IUGR. Issues identified in the review were in relation to variances in definition of IUGR, small sample sizes and the lack of technological consistency across studies.

**Conclusion:**

NIEA shows promise as an adjunct surveillance tool in fetal diagnostics for IUGR. Larger prospective studies should be directed towards establishing reliable parameters with a focus on uniformity of IUGR definition, technological consistency and the individualisation of NIEA parameters.

**Electronic supplementary material:**

The online version of this article (10.1186/s12884-019-2357-9) contains supplementary material, which is available to authorized users.

## Background

Approximately 11% of all pregnancies in developed nations and up to 23.8% of pregnancies in developing nations are complicated by intra uterine growth restriction (IUGR) [1, 2].

IUGR refers to the inability of a fetus to achieve its genetic growth potential in utero [3, 4]. Its aetiology may entail either maternal, fetal and placental factors, acting individually or in tandem, and is clinically differentiated into both early (< 32 weeks of gestation) and late forms (≥32 weeks) [5]. As a phenomenon, IUGR is a risk factor for both perinatal mortality and morbidity [6–8]. Emerging evidence further suggests that exposure to a hostile intra uterine environment can perpetuate through childhood too, with these fetuses being at risk of poor neurodevelopmental outcomes, long term physical growth, cardiovascular and endocrinological disease [9–11].

Clinically, fetal smallness is defined as the weight of fetus being below the 10th weight centile. There however remains some level of difficulty in differentiating isolated fetal smallness from IUGR, particularly at a late gestation, and in managing these findings as well [4, 12]. Recognising this diagnostic dilemma, seminal work has been directed towards developing consensus criteria to accurately identify the IUGR fetus. There however, is still a lack of a gold standard which highlights an urgent need to characterise further tests [4].

In the current context, accurate identification also remains paramount as it would reduce the need for unnecessary interventions and monitoring, which have the benefits of a reduction in healthcare costs whilst negating the medicalisation of essentially normal pregnancies [3].

Non-invasive electrophysiological assessment (NIEA) is an evolving field in fetal diagnostics which is being mooted as a promising tool in fetal surveillance and discriminating between IUGR and normal fetuses. This involves the assessment of temporal and morphological characteristics from the electrophysiological activity of the fetal heart. At present, the technologies by which these can be done non-invasively include non-invasive fetal electrocardiography (NIFECG) and fetal magnetocardiography (FMCG) [13]. An in-depth discourse on these respective technologies can be found in Additional file 1.

### Objectives

Considering the aforementioned issues, the following systematic review was initiated following the PRISMA guidelines. The primary objective was to investigate and define the electrophysiological differences between IUGR and normal or appropriate for gestation age (AGA) fetuses utilising NIEA techniques (either NIFECG or MCG). We also aimed to correlate the utility of the identified NIEA methods with the existing literature in both animal and human models. Furthermore, we also intended to explore the potential physiological mechanisms through which the NIEA parameters enacted their discriminative ability.

### Materials and methods

#### Data sources

For the following review, a systematic search was undertaken independently by VS and JAS across PubMed, Ovid Medline and EMBASE up till 4th May 2018 and searched between 5th of May and 9th of May 2018. The search was subsequently repeated on the 24th of September 2018 and searched between the 25th of September and the 29th of September 2018 by both VS and JAS once again.

Search terms utilised for NIFECG were: “(fetal electrocardiogram AND iugr) OR (foetal electrocardiogram AND iugr) OR (fetal electrocardiogram AND intra uterine growth restriction) OR (foetal electrocardiogram AND intra uterine growth restriction) OR (fetal ecg AND iugr) OR (foetal ecg AND intra uterine growth restriction)” .

Search terms utilised for FMCG were: (magnetocardiogra* AND iugr) OR (magnetocardiogr* AND intra uterine growth restriction) OR (mcg AND intra uterine growth restriction) OR (mcg AND iugr).

Taking technological factors into consideration, limitations were placed on studies that were published after 1980. There were no limitations placed on language. Both human and animal studies were considered for the following review.

The inclusion criteria for articles in this review were those focused on the utilisation of NIEA parameters in comparing the differences between IUGR and normal fetuses antenatally. Technology utilised for the acquisition of the cardiac time intervals (CTIs) were limited to only NIFECG and MCG. Articles were suitable for inclusion regardless of the aetiology of IUGR and clinical presentation (early or late forms). Preference was given to primary articles exploring these changes. Articles of relevance identified from the reference lists of studies searched were suitable for inclusion in the review as well. Articles which were reviews and comprised of case series or case reports were excluded from this review.

Once identified, articles for inclusion in the review were selected in consensus between JAS, VS and AN.

#### Search strategy

**Fig. 1:**
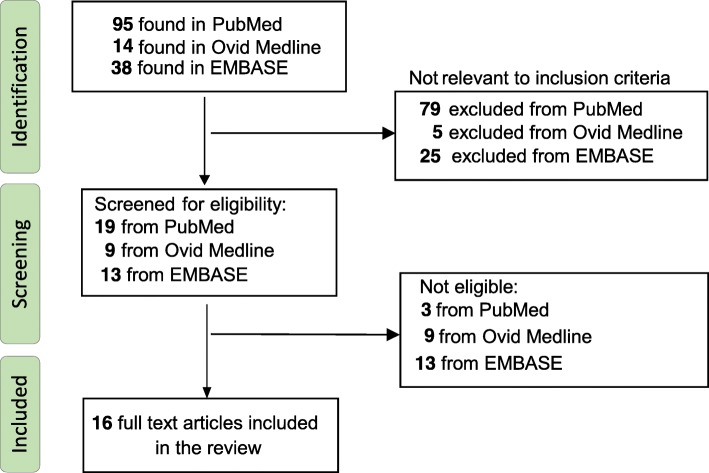
Search strategy for the systemic review

The search strategy for the following study is illustrated in Fig. 1 based on the updated search done on 25th of September 2018.

#### Data collection process

Data for the following study was extracted manually for analysis by VS and JAS. Due to the heterogeneity of the studied populations, variations in technologies, parameters utilised and end points of the studies, pooling of data for meta-analysis was not possible and not considered appropriate. As such, a narrative approach was followed for the following review and a descriptive and qualitative analysis of the studies was carried out to provide quality assessment.

#### Data items

Data items of interest for the following study were the study design, inclusion criteria for the study, method utilised to acquire signal, duration of signal sampling, signal processing method utilised, NIEA parameters utilised and results of those parameters in evaluating IUGR.

#### Assessment of bias

**Fig. 2:**
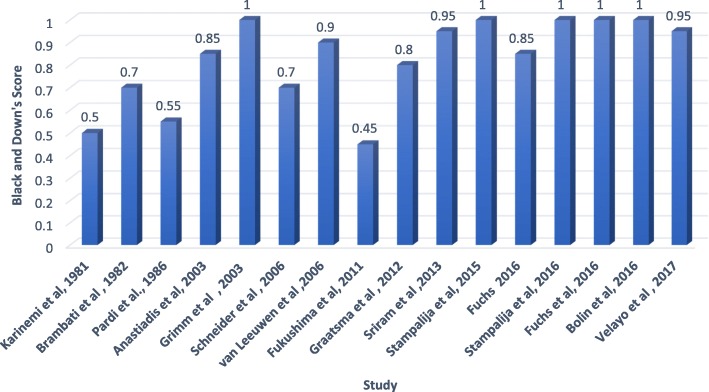
Modified Black and Down’s score for quality of studies included in review (Maximum score achievable is 1.0 or 10 points)

The risk of bias was assessed by VS and RW in consensus utilising the Modified Black and Downs list (Additional file 2). This is a tool which has been used widely to rate the quality of observational studies and is rated on a 10 point scale [14]. Fig. 2 illustrates the score of the studies included in the review. The scores were calculated as the average of scores between VS and RW and the questionnaire is included in the Appendix for reference. The interrater reliability between both raters was very high (ICC = 0.949;*p* = 0.00) for the assessment of bias.

#### Summary measures and synthesis of results

The summary measures for the following study were presented in tabular form for the following review. Importance was given to the NIEA parameters utilised in the studies as well as the signalling methods utilised to obtain them. We further sought to delineate the performance of the NIEA parameters in evaluating IUGR as well.

## Results

### Characteristics of the studies

For this review, a total of 16 studies were suitable for inclusion. Nine studies utilised NIFECG and 7 studies utilised magnetocardiography [15–30]. The characteristics of the studies and their findings have been illustrated in Tables 1 and 2 respectively. The studies were published between 1982 and 2017 and the gestational age of fetuses across studies ranged from 15 to 42 weeks across the studies.

**Table 1:** : Descriptive summary of studies utilising non-invasive fetal electrocardiogram for NIEA analysis

Year	Paper	Study population	Inclusion criteria	Definition of IUGR	Acquisition method	Signal acquisition	Signal processing
1981	Karinemi et al.	Case control study	*N* = 65 from normal pregnancies with no fetal acidosis at birth. *n* = 68 with IUGR. *n* = 84 with post dates pregnancies	BPD < 5%	NIFECG using HP 8030A Cardiotocograph	Recorded manually on magnetic tape and digitalized at a 1000 samples per second)^a^	R-R intervals detected no description provided)^a^. DI sampled over 5 min. Upper rejection limit of 5 bpm was utilised
1982	Brambati et al.	Case control study	*N* = 26 of fetuses suspected to be small for dates	Small for dates by ultrasound or clinical exam (no explanation given) BW < 10% postnatally.	NIFECG- system not specified	N/A	MECG subtraction method utilised. Signal delayed by 180 ms and 50 complexes averaged
1986	Pardi et al.	Prospective cohort study	25 to 41 weeks of gestation. *n* = 68. Fetuses with malformations excluded	AC < 5%	NIFECG- system not specified	N/A	MECG subtraction method utilised. Signal delayed by 180 ms and 50 complexes averaged
2012	Graatsma et al.	Prospective cohort study	20–42 weeks of gestation. Control (*n* = 90),IUGR (n = 30)	EFW ≤10% and BW ≤10% corrected for GA	NIFECG. Monica AN24. 5 electrodes utilised	Recorded with a sample frequency of 1000 Hz. Duration = 7 h	In depth analysis available from Pieri et al.^b^ MECG subtraction method utilised. 50 FECG complexes averaged
2015	Stampalija et al.	Case control study	26–34 weeks. IUGR *n* = 22, Control *n* = 37. Singleton pregnancies	AC < 5%	NIFECG. Monica healthcare. 5 electrodes utilised transabdominally	40 min recording. Sample frequency of 900 Hz	MECG subtraction method as filter. FECG complexes averaged over a 2 s window. PRSA applied to entire trace STV measured using Dawes Redman criteria
2016	Stampalija et al.	Case control study	25–40 weeks. IUGR (*n* = 66). Control (*n* = 79) Singleton pregnancies	AC < 5%	NIFECG. Monica healthcare. 5 electrodes utilised transabdominally	37 min.	R-R wave pulse intervals were calculated with an accuracy of 1 ms. MECG subtraction method as filter. FECG complexes averaged over a 2 s window. PRSA applied to entire trace
2016	Fuchs et al.	Prospective case control study	28–42 weeks. IUGR = 93, IUGR with brain sparing = 37 and 324 healthy pregnancies	EFW < 5% and only asymmetrical IUGR considered. BW was < 10% and ponderal index < 10% at birth	NIFECG. KOMPOREL system. 6 electrodes on maternal abdomen,	30 min recording. No further information provided	No information provided
2016	Fuchs et al.	Prospective case control study	28–40 weeks. IUGR with normal CPR (*n* = 110), IUGR with decreased CPR (*n* = 29) and normal pregnancies (*n* = 549)	EFW < 5% and only asymmetrical IUGR considered. BW was < 10% and ponderal index < 10% at birth	NIFECG. KOMPOREL system. 6 electrodes on maternal abdomen,	30 min recording. No further information provided	No information provided
2017	Velayo et al.	Human- case control study	20–34 weeks IUGR *n* = 15, Control *n* = 20. Singleton pregnancies	EFW and AC < 10%	NIFECG 14 electrodes transabdominally	20 min recordings Bipolar recording. Sampling every 1 ms at 1 kHz	1-100 Hz band pass filtering. FECG signal averaging carried out.

A*C* abdominal circumference,*BPD* Bi parietal diameter, *BW* birth weight, *CPR* Cerebro placental ration, *CTI* cardiac time intervals, *EFW* estimated fetal weight, *GA* gestational age, *FECG* fetal electrocardiogram, *HC* head circumference, *MECG* maternal electrocardiogram, *NIFECG* non invasive fetal electrocardiogram, *IUGR* intra uterine growth restriction, *PRSA* phase rectified signal averaging, *SD* standard deviation, *STV* short term variability

^a.^Yeh et al. [54].^b.^ Pieri et al. [66]

**Table 2:** : Descriptive summary of studies utilising Magnetocardiogram for NIEA analysis

Year	Paper	Study population	Gestational Age, Inclusion & Exclusion criteria	Definition of IUGR	Acquisition method	Signal acquisition	Signal processing
2003	Grimm et al.	Case- control study	21–24 weeks. IUGR *n* = 30. Control *n* = 60.	EFW < 5% and confirmed BW confirmed IUGR	31 channel SQUID biomagnetometer	2 min recordings sampling at 1 kHz.	Band pass filters of 0.3 to 500 Hz. Smoothing with Savitzky-Goolay filter. Complex averaging carried out and maternal subtraction method applied ^a^
2003	Anastasiadis et al.	Case control study	34–37 weeks. IUGR (n = 11) Control (*n* = 19)	AC,HC or BPD < 2SD below the average	Single channel biomagnetometer	32 s duration each. Sampling frequency of 256 Hz.	Power spectral analysis with Fast Fournier transform. Band pass filtered with cut off frequency of 0.1–100 Hz
2006	Schneider et al.	Case control study	28–39 weeks. IUGR (*n* = 36) Control (*n* = 29) Exclusion criteria: known cardiac or fetal malformations,	No definition of IUGR	31 channel SQUID biomagnetometer	5 min recording at a sampling rate of 1000 Hz	Filtering band pass between 0.3 -500 Hz. QRS complexes averaged over 256 beats.
2006	Van Leeuwen et al.	Case control study	15–42 weeks IUGR (*n* = 27), Control (*n* = 47). Singleton pregnancies. Arrythmia and CHD excluded	EFW < 10%	230 FMCG recorded from normal foetuses. 29 FMCG recorded from IUGR group, Acquired using 61 or 37 channel biomagnetometer	5 min recordings Sampling at 1 kHz.	Bandpass filters of 1-200 kHz Fetal beats identified on QRS template using 20–30 beats. PQRST averaged over 300 beats for CTI.
2011	Fukushima et al. (Power spectral analysis)	Case control study	28–39 weeks. IUGR (*n* = 12) and controls (*n* = 35).	EFW < 10%	64 channel SQUID biomagnetometer	5 min recordings.	Maternal subtraction method utilised. Means were average over 300–500 beats. Power spectrum in frequency domain derived from maximum entropy method. LF domain 0.01–0.15 Hz. HF domain 0.15–0.4 Hz
2013	Sriram et al.	Case control study	30–38 weeks. IUGR. Only heart rate data for sleep states were analysed	EFW < 10%	151 sensor SQUID array system. *N* = 40 available for analysis. Pattern A was quiet sleep state and pattern B was active sleep state.	Between 10 and 30 min. Sampling rate of 312.5 Hz	Band pass filtered between 0.5 to 50 Hz.Hibert transform approach utilised to identify R waves. Parameters derived from 6 min windows
2016	Bolin et al.	Case control study	33–37 weeks. IUGR (*n* = 21), hypertensive mothers (*n* = 46) and control (*n* = 74). No fetal cardiac pathology or arrythmias.	EFW < 10%	151 sensor SQUID array system.	CTI averaged over 1 min after maternal with maternal subtraction method	Not described

*AC* abdominal circumference, *BPD* bi parietal diameter, *CTI* cardiac time intervals, *EFW* estimated fetal weight, *HC* head circumference, *IUGR* intra uterine growth restriction, *SD* standard deviation. ^a.^ Schneider U et al. [67]

### Qualitative analysis of studies

The studies included in the review were analysed quantitatively for comparison. Tables 1 and 2 provide descriptive summaries of the studies and information in relation to their design for NIFECG and MCG respectively. Tables 3 and 4 provide a summary of the NIEA parameters utilised specifically in each of these studies and a summary of their findings as well.

**Table 3:** Findings of NIEA parameters in studies utilising non-invasive fetal electrocardiogram

Year	Paper	Parameters assessed	Findings
1981	Karinemi et al.	STV using DI	95.4% of NIFECG were successfully acquired. The distribution of DI in the IUGR group was significantly different to the normal cohort (*p* < 0.001). DI had sensitivity of 64% and predictive value of 80% in screening for fetal distress in the IUGR group(*p* < 0.01)
1982	Brambati et al.	QRS	100% of NIFECG were successfully analysed 96.2% of SFD fetuses had QRS duration less then 2SD below the normal values for gestation. QRS duration (in pregnancy) and live birthweight demonstrated a strong relation (r = 0.74, p < 0.001)
1986	Pardi et al.	QRS	100% of NIFECG were successfully analysed. 81.5% of IUGR fetuses had QRS duration less then 2SD below the normal values for gestation. QRS duration (in pregnancy) and live birthweight demonstrated a linear relation (r = 0.69, p < 0.001) QRS values >4SD below normal were related with abnormal CTG, low APGAR and perinatal deaths
2012	Graatsma et al.	FHR PRSA- AC/DC STV	STV increased in early gestation with stable 3rd trimester values AC and DC remained constant during pregnancy irrespective of gestation. STV abnormal in 16% of the IUGR fetuses AC and DC abnormal in 36 and 40% of IUGR Z scores for IUGR fetuses for STV, AC and DC were lower by 1.0SD, 1.5SD and 1.7SD respectively in comparison to the controls [mean of z- scores, 0;SD-1, (*p* < 0.0001)] In IUGR group, AC and DC z scores were lower than STV scores. When STV z score was utilised with AC and/or DC Z-scores, the findings of deviation became more accentuated
2015	Stampalija et al.	PRSA STV	Significantly lower AC and DC in IUGR vs controls (p < 0.05) for any T ≥ 5 values AUC for AC [0.63 (95% CI 0.47–0.78)–0.87 (95% CI 0.77–0.96)] and DC [0.64 (95% CI 0.48–0.79)–0.89 (95% CI 0.81–0.98)] STV significantly lower between IUGR and controls (8.6 ± 2.4vs 11.1 ± 2.6 ms. *P* = 0.001). AUC for STV 0.77 (95% CI 0.65–0.90). AUC for PRSA significantly outperformed STV
2016	Stampalija et al.	PRSA- AC/DC at T9	AC and DC at T9 were significantly lower in IUGR vs controls after adjusting for GA [OR = 2.1, 95%CI 1.5–3.0 and OR = 0.5 95%CI 0.36–0.68, *p* < 0.001) AC and DC at T9 were higher for IUGR with brain sparing vs those without brain sparing (OR = 1.8, 95%CI 0.97–3.4, *p* = 0.06 and OR = 0.5 95%CI 0.30–0.98, *p* = 0.04)
2016	Fuchs et al.	T/QRS ratio STV FIGO classification of CTG- normal, suspicious and pathological	STV in normal pregnancies (9.08 ± 3.91) were significantly different (*p* < 0.05) from IUGR with brain sparing (11.33 ± 1.38) and IUGR without brain sparing (10.16 ± 4.98) T/QRS values were all below the cut off for abnormal results across all groups Highest average T/QRS ratio (> 0.3) seen in IUGR with brain sparing regardless of FIGO classification of CTG No correlation found between T/QRS ratio and FIGO classification of CTG
2016	Fuchs et al.	T/QRS ratio	Regression did not show any significant differences between groups in relation to GA and T/QRS ratio. T/QRS ratios demonstrated significant differences between IUGR group with reduced CPR and normal CPR (p < 0.001) When using the maximum values and maximum – minimum values, the regression line descends in group with normal CPRs but rises in group with reduced CPR.
2017	Velayo et al.	QT, RR, QRS, ST,PR and PQ intervals. QTc, PR/RR and HR	100% of PQRST were recognised. Both QT and QTc parameters were significantly prolonged (p < 0.05). QT > 267.99 has a sensitivity of 80.0% and a PPV of 40% for IUGR. QTc > 0.43 had a sensitivity > 86.7% and PPV of 40.6%.

*AC* acceleration component, *CTG* cardiotocogram, *CPR* cerebroplacental ratio, *DC* deceleration component, *DI* differential index, *IUGR* intra uterine growth restriction, *NIFECG* non-invasive fetal electrocardiogram, *PRSA* phase rectified signal averaging, *SD* standard deviation, *SFD* small for dates, *STV* short term variability

**Table 4:** Findings of NIEA parameters in studies utilising magnetocardiography

Year	Paper	Parameters assessed	Findings
2003	Grimm et al.	P wave, PR interval, QRS complex and QT interval	P and QRS detected in 100%. T waves detected in 95% of fetuses. P wave and QRS complex length demonstrated significant correlation with GA in AGA fetuses (r = 0.222/0.318, p < 0.05). No correlation was seen in IUGR group. QRS duration was longer in IUGR group vs control group (*p* = 0.009)
2003	Anastasiadis et al.	HRV measured through: LF and HF	LF and HF components were significantly lower in IUGR group (*p* < 0.001). Increased LF tone in IUGR fetuses indication of increased sympathetic tone. HF tone was increased in IUGR as well
2006	Schneider et al.	HRV measured through: SDNN, RMSSD, LF, HF, LF/HF ratio, TP, complexity of AIF decay	SDNN, RMSSD, LF,HF and TP were significantly lower in IUGR group (p < 0.05) No significant difference in LF/HF ratio and AIF decay.
2006	Van Leeuwen et al.	P wave, PQ segment, QRS complex, ST segment, T wave, PR interval and Qt interval	100% of CTIs acquired. IUGR foetuses had shorter depolarization times (P wave, PR interval, QRS complex) and longer ventricular repolarization times (QT interval) then controls. Depolarization times had a positive correlation with fetal weight in the IUGR group [Pwave (r = 0.64), QRS (r = 0.47), *p* < 0.05]
2011	Fukushima et al.	HRV measured through PSA: CV_RR,_ LF/HF ratio	CV_RR_ in controls showed a weak positive correlation with GA (r = 0.32) while IUGR fetuses showed no such trend (r = 0.05). LF/HF ratio showed a moderate positive correlation with GA for controls (r = 0.49) but a weak correlation with IUGR (r = 0.23) No statistically significant changes in LF/HF ratio between normal pregnancy and IUGR groups
2013	Sriram et al.	HRV measured through: PPA,SDNN,RMSSD	IUGR group had lower HRV vs low risk group Using PPA – Both HRV in state A and B were lower (36 and 49% lower, *p* < 0.005) Using SDNN- Both HRV in State A and B were lower (19 and 49%, *p* < 0.005) RMSSD -not statistically significant
2016	Bolin et al.	P wave, PR interval, QRS complex and RR interval	Mean P and PR interval were significantly lower in IUGR group then normal pregnancies (*p* < 0.05) when normalised to 35 weeks of gestation

*AGA* appropriate for gestational age, *AIF* autonomic information flow, *CV*_*RR*_ coefficient of R-R variability, *GA* gestational age, *IUGR* intra uterine growth restriction, *HF* high frequency, *HRV* heart rate variability, *LF* low frequency, *PPA* phase plane area, *RMSSD* root mean square difference of successive heart rates, *SDNN* standard deviation of heart rate, *TP* total power

### Variation in definition of IUGR

There was a variety of definitions utilised for IUGR as inclusion criteria across the studies included in the review. Some adopted an ultrasonographic measure of estimated fetal weight below the 5th centile while others utilised a measure below the 10th centile. Other studies utilised isolated ultrasound parameters such as the Bi- parietal diameter, head circumference or abdominal circumference as part of their inclusion criteria. Finally, two studies utilised post-delivery birth weights and the ponderal index as inclusion criteria for their study. The various definitions can be sourced in Tables 1 and 2 respectively.

### NIEA parameters

The findings of the review in terms of the NIEA parameters and their utility in evaluating IUGR are presented in tabular form in Tables 3 and 4.

### A. P wave duration

Utilising MCG*, Bolin* et al. demonstrated a significantly shorter P wave duration in the IUGR group in comparison to the AGA group (66.4 vs 76.2 ms; *p* = 0.001, 29]. These findings however, were isolated and not mirrored by any other studies in this review. *Van Leeuwen* et al. noted that the P wave duration was non-significantly shorter in the IUGR fetuses in comparison to the AGA fetuses. They also noted that these changes were more apparent in female in comparison to male fetuses [26]. *Grimm* et al. did not demonstrate any discriminative ability of the P wave duration in differentiating the IUGR fetuses from AGA fetuses. They also reported a significantly prolonged PR interval in their AGA which was not evident in their IUGR group [20]. Utilising NIFECG, *Velayo* et al. demonstrated no discriminative ability for the P wave duration in differentiating IUGR from AGA [27].

In summary, the P wave duration did not prove useful in differentiating between IUGR and AGA fetuses.

### B. PR interval

*Bolin* et al. found PR intervals in the IUGR fetuses to be significantly shorter than that of AGA fetuses. Again, these findings were isolated and not mirrored by any other studies [29]. *Van Leeuwen* et al., utilising MCG, noted that the PR interval was non-significantly shorter in the IUGR fetuses in comparison to the AGA fetuses. It however, did not discriminate between the groups. Similarly, *Grimm* et al. did not identify any discriminative ability for the PR interval. Utilising NIFECG, *Velayo* et al. demonstrated no discriminative ability for the PR interval in differentiating IUGR from AGA [27].

In summary, the PR interval did not prove useful in differentiating between IUGR and AGA fetuses.

### C. QRS duration

The following studies demonstrated significantly shortened QRS durations in fetuses with IUGR in comparison to AGA fetuses [16, 20, 22, 26]. Both *Brambati* et al. and *Pardi* et al. demonstrated that 96.2 and 81.5% of IUGR fetuses respectively had a QRS duration less then 2SD below the normal values for gestation. *Van Leeuwen* et al. mirrored these findings male fetuses only in their MCG study [26]. *Grimm* et al. in contrast paradoxically demonstrated a significantly longer QRS duration in the IUGR group in comparison to the AGA [20]. *Velayo* et al. and *Bolin* et al demonstrated no discriminative ability for the QRS duration in differentiating IUGR from AGA [27, 29].

In summary, the utility of the QRS duration remains equivocal in differentiating IUGR from AGA fetuses.

### D. QT interval

Utilising NIFECG, *Velayo* et al. showed significant discriminative ability for the QT interval in distinguishing between IUGR and AGA fetuses. Utilising a QT value of ≤267.99 provided a sensitivity of 80.0% and specificity of 10% with a positive predictive value (PPV) of 40.0% [27].

*Van Leeuwen* et al. and *Grimm* et al. did not demonstrate any discriminative ability for the QT interval utilising MCG [20, 26].

As such, the utility of the QT interval remains equivocal in differentiating IUGR fetuses.

### E. T/QRS ratio

*Fuchs* et al. utilising NIFECG demonstrated the utility of the T/QRS ratio in evaluating IUGR. Utilising multivariate regression analysis, they demonstrated the utility of maximum, and difference between maximum and minimum T/QRS values in differentiating between IUGR fetuses with normal and abnormal cerebroplacental ratios [17]. They were however unable to replicate these results in their subsequent study as the maximum and mean T/QRS values were all below the cut-off level for abnormal levels [17, 18].

As such, the T/QRS ratio remains equivocal in differentiating IUGR fetuses.

### F. Short term variability (STV)

All studies utilising NIFECG to examine the efficacy of STV, demonstrated its discriminative capacity between IUGR fetuses and AGA fetuses. *Stampalija* et al. demonstrated lower STV levels in IUGR fetuses in comparison to AGA fetuses [25]. Similar findings were mirrored by *Graatsma* et al. when utilising z scores for comparison and by *Karinemi* et al. when utilising the differential index with a cut-off level below the 1st centile of normal derived from controls in their study [19]. The STV values determined by *Fuchs* et al. however, were all above the commonly used cut-offs for pathological levels. In addition, they paradoxically demonstrated that the STV values were higher in IUGR fetuses [18] .

As such, the utility of STV appears promising for differentiating IUGR from AGA fetuses.

### G. Long term variability (LTV)

In assessing the LTV, a variety of signal processing methods were utilised. These include:

### H. Phase rectified signal averaging (PRSA) - AC/DC analysis

All studies utilising PRSA analysis demonstrated significant differences between IUGR and AGA fetuses when utilising NIFECG. *Graatsma* et al. demonstrated lower AC/DC z scores in the IUGR fetuses in comparison to the controls. Importantly, they found that the screening potential of low AC/DC values was more accentuated when combining <−1SD z scores of STV to the algorithm [19]. *Stampalija* et al. in both their studies demonstrated the significantly lower AC/DC values in their IUGR groups. Of note is the finding of PRSA analysis significantly outperforming STV in their study when utilising receiver operator characteristic (ROC) curves [24, 25].

As such, the utility of PRSA appears promising in differentiating IUGR from AGA fetuses.

### I. Other techniques

*Anastasiadis* et al., *Sriram* et al.*, Fukushima* et al and *Schneider* et al all utilised a variety of signal processing techniques to measure LTV utilising MCG. *Anastasiadis* et al. and *Schneider* et al. demonstrated significantly lower low and high frequency components in analysis of LTV for IUGR fetuses in comparison to the controls using power spectral analysis [15, 28]. Both *Schneider* et al. and *Fukushima* et al. however could not ascribe any discriminative ability to the utilisation of the LF/HF ratio. *Sriram* et al. and *Schneider* et al utilised several methods to measure LTV including traditional heart rate variability measurements such as SDNN and Root Mean Square of Successive Differences (RMSSD), as well as non-linear methods, such as phase plane analysis (PPA). Both studies noted that the Standard Deviation of Normal to Normal (SDNN) was significantly lower in the IUGR fetuses in comparison to the AGA ones. The results for the (RMSSD) however were conflicting between both studies. Amidst these findings, the discriminative ability of phase plane area (PPA) analysis in particular bears mention as it was able to capture the LTV changes in both quiet and active states of the fetus [23, 28].

Given the findings, the LTV analysis techniques appear promising in evaluating IUGR.

## Discussion

### NIEA parameters

For the following discussion, attempts were made to correlate the findings of our review with the existing literature in both animal and human models. The focus was to draw parallels with the findings from other studies utilising either the CTG, NIFECG or invasive electrocardiography to examine the differences between IUGR and AGA fetuses for the NIEA parameter of interest .

### Cardiac time intervals (CTIs)

CTI analysis utilises a variety of automated computational methods to extract the PQRST waveforms from both the NIFECG and MCG for analysis. Broadly speaking, these encompass signal detection, enhancement, waveform detection and techniques for signal enhancement. These techniques however are beyond the scope of the review but can be accessed from previous work by our group [31].

**Fig. 3:**
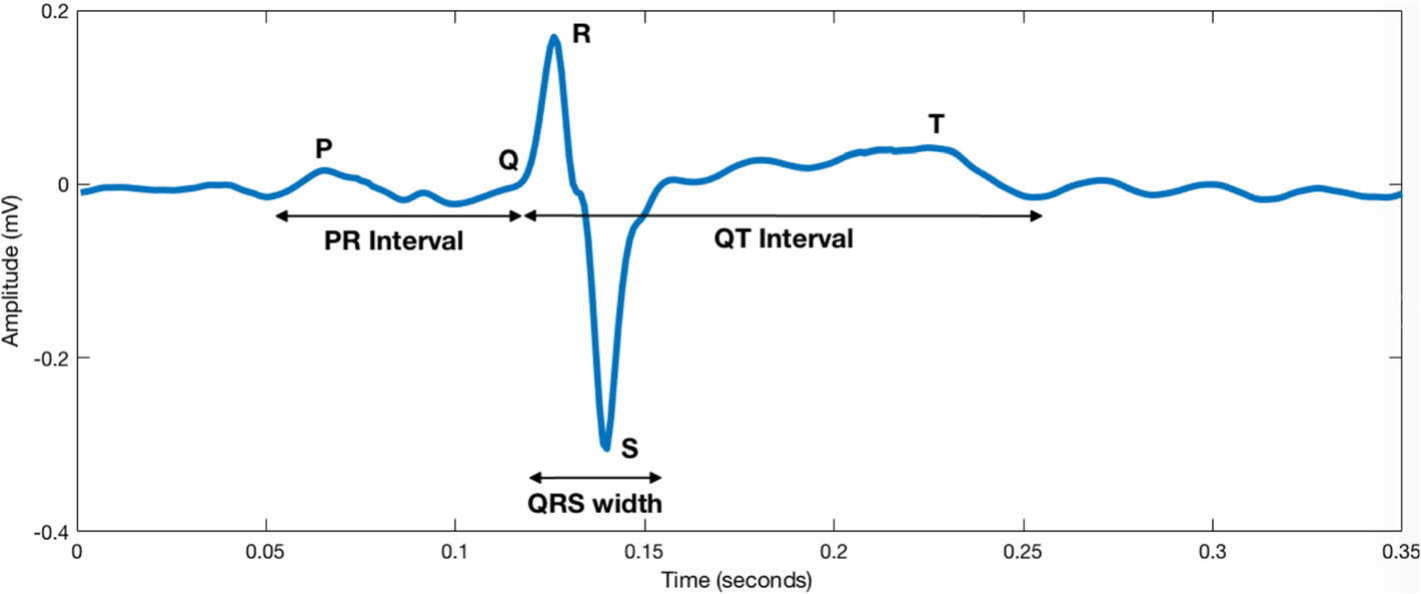
Cardiac time intervals illustrated on a fetal ECG beat. The following beat was extracted from the Physionet database. This was aresult of 10 averaged beats after the maternal ECG was cancelled.

Morphologically, the FECG and MCG are similar to the adult ECG and contain the P wave, QRS complex and T wave as illustrated in Fig. 3. The temporal intervals of significance include the P wave duration, PR interval, QRS duration, QT interval and T/QRS ratio. Information regarding these parameters can be sourced from previous work by our group [31] .

### A. P wave and PR interval

There is no evidence in the literature to which has demonstrated significant differences between the P wave and PR interval between IUGR and AGA fetuses which is in line with the findings of this review.

### B. QRS duration

In examining the literature, the study by *Brambati* et al. utilising NIFECG deserves consideration. In their study on pregnancies afflicted by rhesus immunization, fetuses with cardiac hypertrophy and compensation displayed a QRS duration more than 4SD above the normal mean value. This allowed for improved detection of decompensating fetuses and those at risk of a worse prognosis [32].

The equivocal findings of this review for the QRS duration may be related to its weakly discriminative ability to screen for ventricular hypertrophy which is seen in a subset of IUGR fetuses with cardiac remodelling. As such, the QRS duration may have a yet uncovered role in distinguishing between IUGR and AGA fetuses.

### C. QT interval

There is, however, no evidence in the literature describing differences in the QT interval between IUGR and AGA fetuses.

Interestingly, *Velayo* et al., utilising NIFECG, demonstrated shortening of the QT interval in the recipient fetus in twin -to- twin- transfusion syndrome (TTTS). Given the diastolic dysfunction that these fetuses experience, one may hypothesize that this may signify deteriorating ventricular performance in the fetus [33].

In the context of the equivocal findings for the QT interval in this review, its utility in IUGR evaluation is worth exploring as well.

### D. T/QRS ratio

The T/QRS ratio is calculated by comparing the amplitude of the T wave with the amplitude of the QRS complex. This ratio was proposed as a quantitative measure of the T wave amplitude to eliminate issues with signal gain and amplification from transabdominal electrodes. The T/QRS ratio is known to be affected by myocardial hypoxia and may serve as a marker for it [34–37].

*Widmark* et al. in their study with guinea pigs utilising the fetal scalp electrode (FSE) utilised the T/QRS ratio to differentiate between growth restricted guinea pigs and controls. They too however, found no utility for the T/QRS ratio in differentiating between both groups [36].

It must be mentioned however, that the T/QRS ratio is not one which is normally distributed and may require individualisation for clinical application. This could possibly explain inconsistencies in the marker when cut-offs were utilised for detection of fetal hypoxia. ***Van*** *Wijngaarden* et al. instead proposed individualised cut-offs to be calculated per patient and found discriminative ability for hypoxia when there was an increase over the 97.5th and 99.5th centile for 2 consecutive minutes [38].

In the context of these findings, there may yet be a potentially unproven role for T/QRS ratios in distinguishing between IUGR and AGA fetuses. These values, however, may need to be individualised to the fetus and carried out temporally in order to be of value.

### E. Heart rate variability (HRV)

#### STV

The STV is a computerised approximation of a physiological process which interrogates the interplay between the sympathetic, parasympathetic and peripheral chemoreceptors [39]. It measures the beat to beat variation of the fetal heart which cannot be discerned from visual inspection of the CTG alone [39, 40].

In evaluating the discriminating role of STV, several animal studies remain informative. *Widmark* et al., utilising a guinea pig model, noted STV to be lower in IUGR fetuses in comparison to their AGA counterparts while using invasive electrocardiography (1.96 ± 0.18 vs 3.41 ± 0.32; *p* < 0.02). Similarly, in a study by *Morutsuki* et al. utilising chest electrodes and the Dawes Redman criteria, sheep fetuses with IUGR had a 20% significantly reduced STV in comparison to AGA fetuses (8.8 ± 0.5 vs 10.6 ± 0.5 ms; *p* < 0.05). Likewise, utilising invasive electrocardiography, *Minato* et al. demonstrated STV to be significantly lower in mice with IUGR in comparison to their AGA counterparts (3.31 vs 7.58 ms; *p* = 0.0045) [41].

The available data on STV therefore does seem to suggest that it is consistently lower in IUGR fetuses in comparison to controls of similar gestations and that it may have a yet unfounded role in differentiating between both groups.

### F. Long term variability

#### 1. Classical methods

The long-term variability (LTV) is a commonly used marker on the CTG to assess the interplay between the sympathetic and parasympathetic trunks of the autonomic nervous system of the fetus. It is commonly assessed by clinicians using visual inspection and as such is subject to observer and measurement bias [42, 43]. Classically, methods which have been utilised to quantify this interplay of the fetal cardiac system include the RMSSD and SDNN.

In a recent study utilising computerised cCTG (cCTG), *Stroux* et al. examined the differences between IUGR fetuses from AGA controls using LTV in various parts of their sleep and wake cycles. Their data showed a lower percentage of high variability (active sleep) episodes in fetuses with IUGR compared to controls. In particular, these findings were more pronounced in the gestations ≤34 weeks, where reduced LTV during active sleep states showed an AUC of 74% (70–78%) for distinguishing fetuses with IUGR from AGA fetuses [44].

Another method which is utilised to interrogate this interplay is power spectral analysis (PSA). PSA attempts to quantify the chaotic and dynamic interplay of heart rate variability and attempts to functionally measure the elasticity of the cardiac regulating system as well as the sympthovagal balance. [45, 46] . The low frequency (LF) components are generally thought to correlate with sympathetic tone while the high frequency (HF) components are postulated to correlate with parasympathetic tone and respiratory sinus arrythmia [15, 47].

IUGR fetuses are theorised to have a decreased level of correlation between the HF and LF due to their chronically hypoxic state which in turn negates their ability to compensate to in utero stressors [15]. The study by *Anastasiadis* et al. remains informative in this context.

In line with the findings of the review, the discriminative ability of the HF and LF components also resonate with the study conducted by *Ohta* et al which demonstrated a correlation in IUGR fetuses with LF components and fetal oxygen and pH levels post delivery [47].

#### 2. Novel methods

In recent times however, increasing attention is being directed to non-linear methods to quantify and provide a more physiological measure of the interplay between the parasympathetic and sympathetic trunks of the nervous system and modulation on cardiac functionality [48]. The techniques utilised by studies in this review include:

#### Phase rectified signal averaging (PRSA)

PRSA is an algorithm utilised in signal processing to identify quasi-periodic patterns in a signal which can be masked by the non-stationary nature of the associated signal and noise [49]. It is increasingly being mooted as an alternative measure of STV [50]. This method of signal processing may be better suited to the NIEA signals given its quasi stationary nature. The algorithm measures both the acceleration capacity (AC) and deceleration capacity (DC) of the fetal heart and aims to explore the individualised effects of both arms of the autonomic nervous system (ANS) [51].

Three studies in this review utilised PRSA in evaluating IUGR [19, 24, 25].

The study by *Lobmaier* et al. examined the differences between IUGR fetuses (with and without doppler changes) and AGA fetuses utilising the AC component on cCTG against STV. They noted AAC to have outperformed STV for detection of IUGR. AAC had a PPV and NPV of 90% while STV had a PPV of 71% and NPV of 81%. Furthermore, the area under the curve (AUC) for AC was 97% [95% CI 0.95–1.0] whilst that for STV was 0.85 (95% CI 0.76–0.93). The optimum cut-off level for AC was determined to be 2.4 bpm [52].

As such, PRSA analysis seems promising across several studies and may outperform STV in differentiating between IUGR and AGA fetuses. The available evidence hence supports the findings of the review in its utility.

#### Phase plane area (PPA)

Unlike classical methods of deriving HRV,,PPA examines the relationship of both HF and LF components of heart rate in an interactive manner [23]. Studies in the sheep model have demonstrated the corollary of high frequency components with parasympathetic activity and low frequency components with both sympathetic and parasympathetic synergism [53].

There is however, no evidence in the literature to which has demonstrated significant differences in the DI between IUGR and AGA fetuses.

#### Differential index (DI)

The DI is another method which is utilised to measure fetal hear rate variability. It measures the variability of the coefficients of variation of the between successive R-R intervals and its measurement is not time sensitive [54].

There is no evidence in the literature to which has demonstrated significant differences in the DI between IUGR and AGA fetuses.

#### Cardiovascular adaptations in IUGR

The NIEA changes encountered in IUGR fetuses are thought to occur as a result of the functional and morphological cardiac changes as well as the autonomic dysregulation which occur in these fetuses [55, 56]. These mechanisms are further expounded in Additional file 3.

### Limitations of current technologies

The findings of this review have identified several NIEA parameters which demonstrate utility in distinguishing between IUGR and AGA fetuses. Available monitoring modalities however, are limited in their ability to acquire this data.

#### A. Cardiotocography

CTG produces an approximation of the fetal heart rate using autocorrelation techniques which compare and average it against the previous doppler waveforms [31, 57] . Due to this autocorrelation process however, the FHR obtained via CTG does not contain true beat to beat variability [31, 58, 59]. Furthermore, the signal obtained is of a lower resolution and is subject to various factors such as maternal expulsion efforts, uterine contractions and ventricular ectopics too [25]. This influences the readings obtained for:

#### CTI data

CTG is unable to capture any CTI data which precludes any temporal or morphological analysis of the FECG complex [31].

#### STV

*Seliger* et al. compared the STV obtained from cCTG against that obtained from MCG and NIFFECG and noted a loss of temporal information when the STV was calculated from cCTG based heart rate data [60]. This was further explored by ***Wretler*** **et al**. who noted a discrepancy between the STV calculated from the FSE when compared to cCTG [difference in STV = 0.0 msec (R -2.9 – 5.5) [61]] .

#### Power spectral analysis

Power spectral analysis requires both LF and HF to allow for accurate signal processing. CTG only provides the LF components for processing thereby precluding its use [47].

The ability of the NIFECG to successfully acquire and process these signals however, is dependent on an adequate number of electrodes and a sampling rate of approximately 1000 Hz, thereby highlighting a potential technological limitation [62, 63].

#### PRSA analysis

Both arms of the ANS operate on separate frequency domains [sympathetic component (0.04–0.15 Hz) and parasympathetic (0.15–1.0 Hz)]. The AC and DC utilise the fetal R-R interval (separation between an R peak and the following R peak) from the FECG as a key reference point for calculation. CTG however does not acquire or report any R-R intervals which would affect the accuracy of the derivation [25, 64].

#### B. Fetal scalp electrode

The FSE generates FHR by identifying the R-R interval from the direct FECG signal. This method allows for assessment of all NIEA parameters. It is however limited by its invasive nature where it needs to be directly applied to the fetal scalp and requires the rupture of membranes. In addition, its utility antenatally is limited and it is predominantly utilised in labour. Furthermore, it has risks associated with it such as injury to the fetal scalp and the vertical transmission of infections [65].

### Avenues for future research direction

This systematic review highlights that further large-scale prospective studies are required to investigate the value of NIEA techniques in differentiating between IUGR form AGA fetuses. At present, the evidence for utilising non-invasive modalities with accurate NIEA parameters, such as MCG and NIFECG, remains limited. Tables 1, 2, 3 and 4 could be utilised as a reference matrix to design these studies. The following issues should be taken into consideration when designing and executing the trials:

#### Uniformity in definition of IUGR

We propose that the biometric parameters of estimated fetal weight and abdominal circumference < 10% be utilised to promote uniformity across studies. Doppler indices may be utilised to further differentiating between fetuses with features of cardiovascular compensation and cerebral redistribution.

#### Utilisation of all NIEA parameters for comparison across groups

We suggest that the following NIEA parameters be explored in future studies: P wave duration, PR interval, QRS duration, QT interval, T/QRS ratio, LTV and STV. These techniques can be utilised for both the NIFECG and MCG and this will promote meta-analysis of the data. In addition, importance should be directed towards the QRS and QT duration, T/QRS ratio, STV and PRSA analysis (LTV) which show promise in their utility for IUGR evaluation. We also suggest the addition of cardiac circumference to thoracic circumference (CC/TC) ratio to explore the FECG changes related to cardiac size in an attempt to correlate this with the postulated cardiovascular changes which occur in these fetuses [27].

#### Data analysis of NIEA parameters

During data analysis, researchers should consider if the parameters obtained are normally distributed or would require individualisation. As demonstrated with T/QRS ratio as well as STV, the screening potential of certain parameters may be optimised through individualisation.

#### Technological shortcomings

We would like to highlight the technological shortcomings when utilising NIFECG between the 28th to the 32nd week of gestations given the presence of vernix caseosa in the fetus. This is known to cause signal attenuation, and this could cause false positives and false negatives to be introduced into the calculation of the NIEA parameters. This can be overcome by utilising an increased number of leads in these gestations and employing greater signal amplification and utilising more robust de-noising techniques such as adaptive maternal ECG cancellation as well as better noise identification through signal quality metrics [15].

#### Timing of delivery

An additional area of interest which is worth exploring when the NIEA parameters have been adequately defined would be the timing of delivery in IUGR fetuses on the basis of the various NIEA parameters.

### Limitations

#### Small sample size

The size of the samples of the studies utilised in these studies were considerably small and this is a considerable limitation in extrapolating their findings to the general population.

#### Quality of evidence

Being predominantly observational in nature, the studies included in this review were at high risk of bias due to their inherent design and methodological shortcomings. This should be kept in mind when interpreting the findings.

#### Technological consistency

The signal acquisition and processing methods employed across studies were varied. The averaging of heart beats or width of window utilised for CTI extraction in NIEA analysis in particular could have introduce measurement bias. We propose window sizes less than 5 s in duration for the averaging as well given the quasi stationary nature of FHR and importance of heart rate variability in the fetus.

Even considering the limitations however, it must be highlighted that the following studies have demonstrated promise and have outlined the scientific merit in further evaluating their ability to effectively screen for IUGR.

## Conclusion

NIEA analysis is in the early phase of adoption and the literature remains sparse in its utility for IUGR evaluation. The findings of this review however, suggest that there may be a role for these techniques in improving the detection of IUGR. This is hypothesised to be in relation to the cardiovascular and autonomic adaptations which occur with IUGR fetuses. In particular, the QRS and QT duration, T/QRS ratio, STV and PRSA analysis (LTV) show promise in augmenting the detection algorithms. Issues that researchers should be aware of include the definition utilised for IUGR in their studies as well as the signal acquisition and processing methods utilised. Individualisation of parameters, especially the T/QRS ratio and STV, may prove further discriminative potential.

## Additional files

Additional file 1: Information relating to technologies utilised to capture NIEA parameters. (DOCX 41 kb)

Additional file 2: Sample of Modified Black and Down's criteria utilised for assessment of bias. (DOCX 13 kb)

Additional file 3: Cardiovascular adaptations in IUGR. (DOCX 40 kb)

## Data Availability

All data generated or analysed during the study is included in this published article.

## Abbreviations

AGA: Appropriate for gestation age fetuses
CTI: Cardiac time intervals
FECG: Fetal electrocardiogram
IUGR: Intra uterine growth restriction
LTV: Long term variability
MCG: Fetal magnetocardiogram
MECG: Maternal ECG
NIEA: Non-invasive electrophysiological assessment
NIFECG: Non-invasive fetal electrocardiogram
SGA: Small for gestational age fetus
SNR: Signal-to-noise ratio
STV: Short term variability
