# Supplementary material for: A systematic review on the utility of non-invasive electrophysiological assessment in evaluating for intra uterine growth restriction
Source: BMC Pregnancy Childbirth. 2019 Jul 5;19:230. doi: 10.1186/s12884-019-2357-9 (PMC6610904; doi:10.1186/s12884-019-2357-9)
Supplement: Supplementary file 1 — Information relating to technologies utilised to capture NIEA parameters. (DOCX 41 kb) [file 12884_2019_2357_MOESM1_ESM.docx]

**Supplementary Information A**

**1. Non invasive electrophysiological assessment technologies**

- 1. **Non-invasive fetal electrocardiography**

The NIFECG is an emerging technology which obtains fetal heart rate (FHR) information from the maternal abdomen and provides real time visualisation of the fetal electrophysiological process. The signal is obtained using surface electrodes, similar to those utilised in adult electrocardiography. Previously, technological limitations had been the main limiting factor to its uptake. The predominant issues encountered were in relation to the low signal-to-noise ratio (SNR) of the fetal electrocardiogram (FECG) signal [due to its low electrical amplitude (1/50^th^ that of the maternal ECG signal)] which is buried within the large maternal ECG (MECG) signal and background noise and the attenuation posed to signal conduction by the vernix caseosa [1-3]. Nevertheless, recent signal processing techniques and data processors have sparked a renaissance into its utility which has helped develop more consistent non-invasive acquisition methods[1, 3].

FECG has drawn increasing attention over recent years given its potential to[3, 4]:

1. Provide electrophysiological data, such as cardiac time intervals (CTI) and true beat to beat variability [5, 6].
2. Reduce the occurrence of fetal and maternal heart rate confusion [6, 7]
3. Allow for better FHR monitoring in women with a high Body Mass Index[6, 8, 9]
4. Be utilised antenatally from gestations as early as 20 weeks and in labour[10, 11]
5. Provide further morphological information from the FECG, such as the ST levels and PR interval which could further aid in obstetric decision making[12].
6. Provide concomitant accurate monitoring of uterine contraction through the utilisation of the electrohysterogram [13].
7. Provide data on fetal movements and fetal orientation in labour[1].
   1. **Fetal magnetocardiography (MCG)**

MCG is an alternative non-invasive technology which can help delineate cardiac electrophysiology through the utility of highly sensitive sensors which can detect the fetal heart signals approximately 1 pT separate to the maternal signals which are greater than 10pT. The Superconducting Quantum Interference Device (SQUID) systems utilised for heart signal detection are highly sensitive to magnetic flux density and allow for significant signal noise reduction. As a result, the fetal cardiac signals are of higher quality than that obtained via the NIFECG due to the relatively low impact of signal interference from the maternal signal. As such, fetal electrophysiology on the FMCG has been visualised from as early as 13 weeks of gestation in the fetus [14]. Furthermore, MCG is also able to record sequential information across varying gestations without issues of attenuation from the vernix caseosa seen with the NIFECG between the gestations of 27 to 32 weeks [3, 15, 16].

Fetal magnetocardiography has been utilised successfully in:

1. Delineating electrophysiological cardiac data, including assessment of the fetal autonomic system, and is the gold standard in measuring fetal CTIs[15, 17]
2. Fetal arrythmia detection[18]
3. Determining true beat to beat heart rate variability [19]

It must be borne in mind however, that the equipment for MCG is expensive and requires adequately trained personnel to be operated. Moreover, additional ongoing costs are required for its utilisation through consumables such as helium and dedicated shielding facilities for the procedure. Furthermore, The SQUID machines lack portability due to their bulkiness. Importantly, ultrasonographic determination of fetal position is required prior to its utilisation for accurate positioning of the sensors [15, 16].

References

- 1. 1. Clifford GD, Silva I, Behar J, Moody GB: **Non-invasive fetal ECG analysis**. *Physiological measurement* 2014, **35**(8):1521-1536.
  2. 2. Symonds EM, Sahota D, Chang A: **The intervals and morphology of the fetal ECG**. In: *Fetal Electrocardiography.* edn.: PUBLISHED BY IMPERIAL COLLEGE PRESS AND DISTRIBUTED BY WORLD SCIENTIFIC PUBLISHING CO.; 2001: 89-107.
  3. 3. Smith V, Arunthavanathan S, Nair A, Ansermet D, da Silva Costa F, Wallace EM: **A systematic review of cardiac time intervals utilising non-invasive fetal electrocardiogram in normal fetuses**. *BMC Pregnancy and Childbirth* 2018, **18**(1):370.
  4. 4. Behar J, Andreotti F, Zaunseder S, Oster J, Clifford GD: **A practical guide to non-invasive foetal electrocardiogram extraction and analysis**. *Physiological measurement* 2016, **37**(5):R1-r35.
  5. 5. Reinhard J, Hayes-Gill BR, Schiermeier S, Hatzmann W, Herrmann E, Heinrich TM, Louwen F: **Intrapartum signal quality with external fetal heart rate monitoring: a two way trial of external Doppler CTG ultrasound and the abdominal fetal electrocardiogram**. *Archives of gynecology and obstetrics* 2012, **286**(5):1103-1107.
  6. 6. Reinhard J, Hayes-Gill BR, Schiermeier S, Hatzmann H, Heinrich TM, Louwen F: **Intrapartum Heart Rate Ambiguity: A Comparison of Cardiotocogram and Abdominal Fetal Electrocardiogram with Maternal Electrocardiogram**. *Gynecologic and Obstetric Investigation* 2013, **75**(2):101-108.
  7. 7. Stampalija T, Signaroldi M, Mastroianni C, Rosti E, Signorelli V, Casati D, Ferrazzi EM: **Fetal and maternal heart rate confusion during intra-partum monitoring: comparison of trans-abdominal fetal electrocardiogram and Doppler telemetry**. *The journal of maternal-fetal & neonatal medicine : the official journal of the European Association of Perinatal Medicine, the Federation of Asia and Oceania Perinatal Societies, the International Society of Perinatal Obstet* 2012, **25**(8):1517-1520.
  8. 8. Euliano TY, Darmanjian S, Nguyen MT, Busowski JD, Euliano N, Gregg AR: **Monitoring Fetal Heart Rate during Labor: A Comparison of Three Methods**. *J Pregnancy* 2017, **2017**:8529816.
  9. 9. Cohen WR, Hayes-Gill B: **Influence of maternal body mass index on accuracy and reliability of external fetal monitoring techniques**. *Acta obstetricia et gynecologica Scandinavica* 2014, **93**(6):590-595.
  10. 10. Pieri JF, Crowe JA, Hayes-Gill BR, Spencer CJ, Bhogal K, James DK: **Compact long-term recorder for the transabdominal foetal and maternal electrocardiogram**. *Medical & biological engineering & computing* 2001, **39**(1):118-125.
  11. 11. Graatsma EM, Jacod BC, van Egmond LA, Mulder EJ, Visser GH: **Fetal electrocardiography: feasibility of long-term fetal heart rate recordings**. *BJOG : an international journal of obstetrics and gynaecology* 2009, **116**(2):334-337; discussion 337-338.
  12. 12. Reinhard J, Hayes-Gill BR, Yi Q, Hatzmann H, Schiermeier S: **Comparison of non-invasive fetal electrocardiogram to Doppler cardiotocogram during the 1st stage of labor**. *Journal of perinatal medicine* 2010, **38**(2):179-185.
  13. 13. Reinhard J, Hayes-Gill BR, Schiermeier S, Hatzmann W, Herrmann E, Heinrich TM, Louwen F: **Intrapartum signal quality with external fetal heart rate monitoring: a two way trial of external Doppler CTG ultrasound and the abdominal fetal electrocardiogram**. *Archives of gynecology and obstetrics* 2012, **286**(5):1103-1107.
  14. 14. Sriram B, Mencer MA, McKelvey S, Siegel ER, Vairavan S, Wilson JD, Preissl H, Eswaran H, Govindan RB: **Differences in the sleep states of IUGR and low-risk fetuses: An MCG study**. *Early human development* 2013, **89**(10):815-819.
  15. 15. Kiefer-Schmidt I, Lim M, Wacker-Gussmann A, Ortiz E, Abele H, Kagan KO, Kaulitz R, Wallwiener D, Preissl H: **Fetal magnetocardiography (fMCG): moving forward in the establishment of clinical reference data by advanced biomagnetic instrumentation and analysis**. *Journal of perinatal medicine* 2012, **40**(3):277.
  16. 16. Lewis MJ: **Review of electromagnetic source investigations of the fetal heart**. *Medical Engineering & Physics* 2003, **25**(10):801-810.
  17. 17. Fukushima A, Nakai K, Kanasugi T, Terata M, Sugiyama T: **Assessment of fetal autonomic nervous system activity by fetal magnetocardiography: comparison of normal pregnancy and intrauterine growth restriction**. *Journal of pregnancy* 2011, **2011**:218162.
  18. 18. Stingl K, Paulsen H, Weiss M, Preissl H, Abele H, Goelz R, Wacker-Gussmann A: **Development and application of an automated extraction algorithm for fetal magnetocardiography - normal data and arrhythmia detection**. *Journal of perinatal medicine* 2013, **41**(6):725-734.
  19. 19. Hrtankova M, Biringer K, Sivakova J, Sumichrastova P, Lukac P, Danko J: **[Fetal magnetocardiography: a promising way to diagnose fetal arrhytmia and to study fetal heart rate variability?]**. *Ceska gynekologie* 2015, **80**(1):58-63.
