# Supplementary material for: A systematic review on the utility of non-invasive electrophysiological assessment in evaluating for intra uterine growth restriction
Source: BMC Pregnancy Childbirth. 2019 Jul 5;19:230. doi: 10.1186/s12884-019-2357-9 (PMC6610904; doi:10.1186/s12884-019-2357-9)
Supplement: Supplementary file 2 — Sample of Modified Black and Down's criteria utilised for assessment of bias. (DOCX 13 kb) [file 12884_2019_2357_MOESM2_ESM.docx]

Supplementary Information B:

Modified Downs and Black checklist for assessing quality of studies

| No. | Question | Yes = 1 | No = 0 |
| --- | --- | --- | --- |
| Reporting | |  |  |
| 1. | Is the hypothesis/aim/objective of the study clearly described? |  |  |
| 2. | Are the main outcomes of the study clearly described? |  |  |
| 3 | Are the characteristics of the patients included in the study clearly described? |  |  |
| 6 | Are the main findings of the study clearly described? |  |  |
| 7 | Does the study provide estimates of the random variability in the data for the main outcomes? |  |  |
| 10 | Have actual probability values been reported (e.g.  0.035 rather than <0.05) for the main outcomes except where the probability value is less than 0.001? |  |  |
| External validity | |  |  |
| 11 | Were the subjects asked to participate in the study representative of the entire population from which they were recruited? |  |  |
| 12 | Were those subjects who were prepared to participate representative of the entire population from which they were recruited? |  |  |
| Internal validity | |  |  |
| 18 | Were the statistical tests used to assess the main outcomes appropriate? |  |  |
| 20 | Were the main outcome measures used accurate (valid and reliable)? |  |  |
