# Supplementary material for: A systematic review on the utility of non-invasive electrophysiological assessment in evaluating for intra uterine growth restriction
Source: BMC Pregnancy Childbirth. 2019 Jul 5;19:230. doi: 10.1186/s12884-019-2357-9 (PMC6610904; doi:10.1186/s12884-019-2357-9)
Supplement: Supplementary file 3 — Cardiovascular adaptations in IUGR. (DOCX 40 kb) [file 12884_2019_2357_MOESM3_ESM.docx]

1. **Cardiovascular adaptations in IUGR**

One of the main mechanisms implicated in IUGR is in relation to placental insufficiency. As placental function progressively deteriorates with increasing gestation, the fetus adapts to this hostile intra uterine environment through several characteristic circulatory adaptations encompassing centralisation of its circulation [1, 2]. Left unabated, this process eventually leads to blood being shunted preferentially to the fetal heart– a phenomenon termed as ‘heart sparing’. It is hypothesised that prolonged exposure to these cardiovascular compensation mechanisms promotes aberrancy in the electrophysiology of the fetus, thereby prompting NIEA changes. [1, 3]. The means through which this is postulated to occur include:

**1.1 Functional and morphological changes**

The study by ***Fouzas et al.*** remains informative in illustrating the impact of morphological and functional changes on fetal heart electrophysiology. In their comparative study of neonates who suffered from IUGR in utero against controls, they demonstrated significant differences in electrophysiology between both groups, especially in relation to ventricular repolarisation patterns [4].

In terms of morphological changes, studies have demonstrated lower fetal heart weight, shorter sarcomere lengths, thinner cardiomyocytes and increased coronary blood flow in IUGR fetuses in comparison to AGA fetuses. These morphological changes are thought to be linked to the cardiac remodelling that occurs in this group of fetuses as an adaptive response to reduce myocardial metabolism demands in a hostile environment [3, 5, 6] [7].

IUGR fetuses have also demonstrated differential cardiac functional changes in comparison to AGA fetuses as well. With worsening intrauterine fetal compromise, IUGR fetuses demonstrated an increased modified – myocardial performance index, b-type natriuretic peptide levels and Troponin I and T levels. This trend was also observed with relation to systolic and diastolic dysfunction in these fetuses as well [8-15].

Cumulatively, these findings allude to a potential basis for the CTI changes encountered in these fetuses.

**1.2 Autonomic dysregulation**

The fetal heart rate is controlled by the synergistic interplay of both the sympathetic and parasympathetic trunks of the autonomic nervous system (ANS), the central nervous system and chronotropic hormones. Clinically this interplay is interrogated through measurement of HRV. In fetuses with IUGR, it is hypothesized that exposure to chronic hypoxemia in utero manifests clinically in the form of cardiac autonomic dysregulation [3, 16, 17].

Evidence of this is illustrated in the literature. Studies utilising cCTG comparing IUGR fetuses to AGA controls have found significantly lower instances of both STV and LTV in the former group [18-20]. In addition, the study by ***Snijders et al*** remains informative in illustrating the progressive deterioration in HRV parameters temporally in IUGR fetuses in tandem with worsening fetal status[21].

These features of autonomic dysregulation have also been demonstrated in newborns who have been subjected to the stresses of IUGR in utero. In the study by ***Galland et al.***, IUGR neonates demonstrated significantly different markers of sympathetic and parasympathetic control in comparison to AGA controls and these changes continued to persist months into postnatal life[22].

Taking these factors of impaired autonomic functioning into consideration, one may hypothesize that it is through these mechanisms that changes in HRV parameters prove useful in differentiating between IUGR and AGA fetuses.

**References**

1. Baschat AA: **Planning management and delivery of the growth-restricted fetus**. *Best practice & research Clinical obstetrics & gynaecology* 2018, **49**:53-65.

2. Khalil A, Thilaganathan B: **Role of uteroplacental and fetal Doppler in identifying fetal growth restriction at term**. *Best Practice & Research Clinical Obstetrics & Gynaecology* 2017, **38**:38-47.

3. Cohen E, Wong FY, Horne RSC, Yiallourou SR: **Intrauterine growth restriction: impact on cardiovascular development and function throughout infancy**. *Pediatric research* 2016, **79**:821.

4. Fouzas S, Karatza AA, Davlouros PA, Chrysis D, Alexopoulos D, Mantagos S, Dimitriou G: **Heterogeneity of ventricular repolarization in newborns with intrauterine growth restriction**. *Early human development* 2014, **90**(12):857-862.

5. Iruretagoyena JI, Gonzalez-Tendero A, Garcia-Canadilla P, Amat-Roldan I, Torre I, Nadal A, Crispi F, Gratacos E: **Cardiac dysfunction is associated with altered sarcomere ultrastructure in intrauterine growth restriction**. *American Journal of Obstetrics & Gynecology* 2014, **210**(6):550.e551-550.e557.

6. A. BA, U. G, L. G, I. R, P. WC, R. HC: **Coronary artery blood flow visualization signifies hemodynamic deterioration in growth‐restricted fetuses**. *Ultrasound in Obstetrics & Gynecology* 2000, **16**(5):425-431.

7. Bauer R, Walter B, Hoppe A, Gaser E, Lampe V, Kauf E, Zwiener U: **Body weight distribution and organ size in newborn swine (sus scrofa domestica) -- a study describing an animal model for asymmetrical intrauterine growth retardation**. *Experimental and toxicologic pathology : official journal of the Gesellschaft fur Toxikologische Pathologie* 1998, **50**(1):59-65.

8. Severi FM, Rizzo G, Bocchi C, D'Antona D, Verzuri MS, Arduini D: **Intrauterine growth retardation and fetal cardiac function**. *Fetal diagnosis and therapy* 2000, **15**(1):8-19.

9. Miyague NI, Ghidini A, Fromberg R, Miyague LLT: **Alterations in Ventricular Filling in Small-for-Gestational-Age Fetuses**. *Fetal diagnosis and therapy* 1997, **12**(6):332-335.

10. Bahtiyar MO, Copel JA: **Cardiac changes in the intrauterine growth-restricted fetus**. *Seminars in perinatology* 2008, **32**(3):190-193.

11. Verburg BO, Jaddoe VW, Wladimiroff JW, Hofman A, Witteman JC, Steegers EA: **Fetal hemodynamic adaptive changes related to intrauterine growth: the Generation R Study**. *Circulation* 2008, **117**(5):649-659.

12. Cruz-Martinez R, Figueras F, Hernandez-Andrade E, Oros D, Gratacos E: **Changes in myocardial performance index and aortic isthmus and ductus venosus Doppler in term, small-for-gestational age fetuses with normal umbilical artery pulsatility index**. *Ultrasound in obstetrics & gynecology : the official journal of the International Society of Ultrasound in Obstetrics and Gynecology* 2011, **38**(4):400-405.

13. Crispi F, Hernandez-Andrade E, Pelsers MM, Plasencia W, Benavides-Serralde JA, Eixarch E, Le Noble F, Ahmed A, Glatz JF, Nicolaides KH *et al*: **Cardiac dysfunction and cell damage across clinical stages of severity in growth-restricted fetuses**. *American journal of obstetrics and gynecology* 2008, **199**(3):254.e251-258.

14. Makikallio K, Vuolteenaho O, Jouppila P, Rasanen J: **Association of severe placental insufficiency and systemic venous pressure rise in the fetus with increased neonatal cardiac troponin T levels**. *American journal of obstetrics and gynecology* 2000, **183**(3):726-731.

15. Nomura RM, Cabar FR, Costa VN, Miyadahira S, Zugaib M: **Cardiac troponin T as a biochemical marker of cardiac dysfunction and ductus venosus Doppler velocimetry**. *European journal of obstetrics, gynecology, and reproductive biology* 2009, **147**(1):33-36.

16. Smith JH, Anand KJ, Cotes PM, Dawes GS, Harkness RA, Howlett TA, Rees LH, Redman CW: **Antenatal fetal heart rate variation in relation to the respiratory and metabolic status of the compromised human fetus**. *British journal of obstetrics and gynaecology* 1988, **95**(10):980-989.

17. Shaw CJ, Allison BJ, Itani N, Botting KJ, Niu Y, Lees CC, Giussani DA: **Altered autonomic control of heart rate variability in the chronically hypoxic fetus**. *The Journal of physiology* 2018, **0**(0).

18. Henson G, Dawes GS, Redman CW: **Characterization of the reduced heart rate variation in growth-retarded fetuses**. *British journal of obstetrics and gynaecology* 1984, **91**(8):751-755.

19. Kikuchi A, Shimizu T, Hayashi A, Horikoshi T, Unno N, Kozuma S, Taketani Y: **Nonlinear analyses of heart rate variability in normal and growth-restricted fetuses**. *Early human development* 2006, **82**(4):217-226.

20. Nijhuis IJ, ten Hof J, Mulder EJ, Nijhuis JG, Narayan H, Taylor DJ, Visser GH: **Fetal heart rate in relation to its variation in normal and growth retarded fetuses**. *European journal of obstetrics, gynecology, and reproductive biology* 2000, **89**(1):27-33.

21. Snijders RJM, Ribbert LSM, Visser GHA, Mulder EJH: **Numeric analysis of heart rate variation in intrauterine growth-retarded fetuses: A longitudinal study**. *American journal of obstetrics and gynecology* 1992, **166**(1, Part 1):22-27.

22. Galland BC, Taylor BJ, Bolton DP, Sayers RM: **Heart rate variability and cardiac reflexes in small for gestational age infants**. *Journal of applied physiology (Bethesda, Md : 1985)* 2006, **100**(3):933-939.
